# Intermittent screening and treatment versus intermittent preventive treatment of malaria in pregnancy: user acceptability

**DOI:** 10.1186/1475-2875-9-18

**Published:** 2010-01-14

**Authors:** Lucy A Smith, Caroline Jones, Rose O Adjei, Gifty D Antwi, Nana A Afrah, Brian Greenwood, Daniel Chandramohan, Harry Tagbor, Jayne Webster

**Affiliations:** 1 Department of Infectious & Tropical Diseases, London School of Hygiene & Tropical Medicine, Keppel Street, London. WC1E 7HT, UK; 2 Department of Community Health, School of Medical Sciences, Kwame Nkrumah University of Science & Technology, Private Mail Bag, University Post Office, Kumasi, Ghana

## Abstract

**Background:**

Malaria in pregnancy is associated with increased risks of maternal and foetal complications. Currently, intermittent preventive treatment (IPT) of malaria during pregnancy with sulphadoxine-pyrimethamine (SP) is recommended by the WHO as part of a package of interventions also including insecticide-treated nets and effective case management. However, with increasing resistance to SP, the effectiveness of SP-IPT has been questioned. A randomized controlled trial (RCT) to investigate the relative efficacy of an alternative strategy of intermittent screening and treatment (IST), which involves a rapid diagnostic test for malaria at scheduled ANC visits and treatment of women only if positive, versus SP-IPT has been conducted in Ashanti region, Ghana. This paper reports on a complementary study investigating the acceptability of the different strategies to women enrolled in the trial.

**Methods:**

Data were collected through twelve focus group discussions with women selected at random from the different arms of the RCT, exploring their experiences and perceptions about antenatal care and their involvement in the trial. Content analysis was used to identify relevant themes to structure the results.

**Results:**

Five main themes emerged from participants' experiences of ANC and the RCT that would influence their acceptability of malaria prevention strategies during pregnancy: health benefits; drugs received; tests received; other services received; and health worker attitude. Their own health and that of their baby were strong motivations for attending ANC, and reported favourably as an outcome of being in the RCT. Women were not always clear on the biomedical function of drugs or blood tests but generally accepted them due to strong trust in the health staff. Home visits by staff and free ITNs as part of the trial were appreciated. Politeness and patience of health staff was a very strong positive factor.

**Conclusions:**

Overall, both intermittent screening and treatment and intermittent preventive treatment appeared equally acceptable to pregnant women as strategies for the control of malaria in pregnancy. The women were more concerned about quality of services received, in particular the polite and patient attitude of health staff, and positive health implications for themselves and their babies than about the nature of the intervention.

## Background

*Plasmodium falciparum *infection in pregnancy is associated with an increased risk of maternal and foetal complications including maternal anaemia and low birth weight [1,2]. The WHO has recommended a package of interventions for preventing and controlling malaria infection in pregnancy (MiP) in endemic areas, which includes the early diagnosis and treatment of malaria, intermittent preventive treatment during pregnancy (IPT) using sulphadoxine-pyrimethamine (SP) and the use of insecticide-treated nets (ITNs) [3].

Currently, SP-IPT has been rated as having the most favourable cost-benefit profile because of its relatively low cost, high compliance and efficacy in reducing maternal anaemia and low birth weight [4,5]. However, implementation of IPT in pregnancy in most settings is limited by social, cultural, economic and operational challenges despite good coverage of antenatal services [6,7]. In addition, resistance to SP has been spreading across sub-Saharan Africa and thus the effectiveness of SP-IPT has been questioned [5]. There are few anti-malarial drugs with plasma half-life comparable to that of SP that could be used to replace SP for IPT in areas with high SP resistance. Furthermore, there is very limited data on the efficacy and safety during pregnancy of alternative drugs [8]. Studies have shown that rapid diagnostic tests (RDT) have a reasonable sensitivity to diagnose malaria in pregnancy [9-11]. Screening for malaria using an RDT during focused antenatal care visits and treating those women who are positive for malaria with an effective combination of drugs (termed intermittent screening and treatment (IST)) appears to be an alternative approach to control MiP in areas with high SP resistance. While some women with placental malaria may be missed by the RDT screening, it can be argued that the proportion of women with placental malaria missed by an RDT would be minimal if all women used an ITN, and that the risk of missed placental malaria would be offset by the reduction in women unnecessarily exposed to anti-malarials of uncertain safety profile.

**Figure 1:**
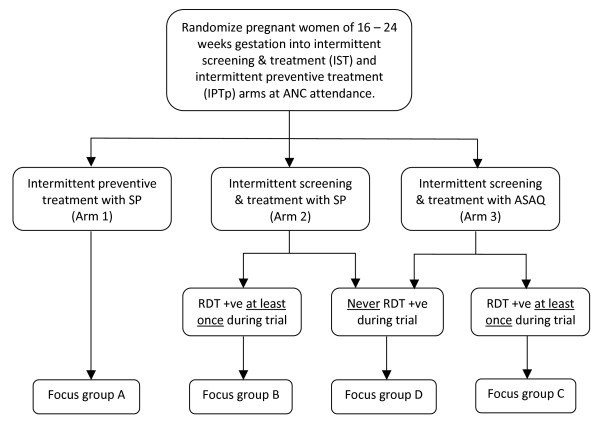
**Schematic diagram to show the allocation of women enrolled in the trial to focus group discussion groups**.

A three-arm, partially-blinded, randomized trial was conducted in the Ashanti Region of Ghana between February 2007 and November 2008 to assess the efficacy of the IST approach compared to SP-IPT. Women in the IST groups were given either SP or artesunate-amodiaquine (AS-AQ) if they had a positive RDT result for malaria and women in the comparison group received SP-IPT and standard case management if they reported with symptoms suggesting clinical malaria (Figure 1). The effect of IST compared to SP-IPT on maternal anaemia, birth weight and foetal outcomes observed in this RCT is reported elsewhere (Tagbor *et al*, personal communication).

However, it has been demonstrated that a highly efficacious intervention given under strict trial conditions rarely translates into an equally effective intervention when implemented under programmatic conditions [12-14]. There are many factors that have the potential to influence the relative implementability of interventions including: the complexity of the interventions [15]; the capacity of the health system and health providers to deliver the interventions at the scale required; and the acceptability of the interventions to providers and users. A series of studies were undertaken to investigate these factors and will be reported elsewhere. Here, the perceptions and experiences of women enrolled in the RCT are presented in order to assess the relative acceptability of IST and IPT to users.

## Methods

The study was conducted during January and February 2009 in Ejisu-Juaben and Sekyere East districts of the Ashanti region of Ghana. These are predominantly rural districts, with the main occupation of the people being subsistence farming. Transmission of malaria is moderately high [16] and occurs throughout the year with peaks during the rains in May-October.

In the Ashanti region 97.4% of women received ANC from a skilled provider at least once during their most recent pregnancy [17], with the majority (69.8%) receiving care from a nurse, midwife or auxiliary midwife, and the remainder receiving care from a doctor (27.6%). All health facilities in the study districts already offer SP-IPT to pregnant women following the Ministry of Health (MoH) guidelines of three doses of SP-IPT. Pregnant women in their second or third trimester diagnosed with malaria are treated with AS-AQ. Counselling and testing for prevention of mother to child transmission of HIV (PMTCT) is also part of core ANC services in Ghana (HIV prevalence amongst adults aged 15-49 in Ghana is 1.9% [18]). Eleven percent of pregnant women in Ashanti region received HIV counselling and testing at ANC and were informed of the results [19]. All maternal health care, including delivery has been free since July 2008, a policy implemented through the National Health Insurance Scheme.

Women for the acceptability study were selected through a combination of purposive and random sampling strategies in order to ensure that experiences of women from each of the three arms of the RCT were represented, and that the views of those women that had had a blood test on each ANC visit but were consistently negative for malaria parasites and so never given any anti-malarials, were summarized adequately. Only women recruited during 2008 were included to limit the length of recall for their experiences of the trial. Women recruited from July 2008 onwards were only recruited if a date of delivery was known. Women were excluded from the sampling frame if the pregnancy was known to have ended in miscarriage or stillbirth, or if a child was known to have died shortly after birth. Of the 1,529 women meeting all other inclusion criteria, 43 (2.8%) were excluded because they had lost their baby.

Women were randomly allocated into groups on the basis of their original RCT study arm or experience in the study. In the RCT, women in the IST arms received a rapid diagnostic blood test at each scheduled ANC visit, drawing blood by a finger prick. Outside of the trial, blood tests were requested by the midwife only if a woman complained of malaria-like symptoms; she would then be sent to the lab and a thick blood film would be taken. The only other diagnostic blood tests routinely carried out at ANC are for HIV, anaemia, sickling and blood group; these are normally only carried out at a woman's first visit to the ANC.

Exploration of the women's opinions on having repeated finger pricks and blood tests at every visit was, therefore, a key objective of the acceptability study. Group A included women who received SP-IPT; Group B involved women in the IST-SP arm with at least one positive RDT; Group C included women in the IST-AS-AQ arm with at least one positive RDT; and Group D involved women in the IST-SP or IST-AS-AQ arms who did not test positive with an RDT at any time during their involvement in the trial (Figure 1).

Data were collected through a series of focus group discussions (FGD). Each FGD was moderated by one member from a team of eight fieldworkers using a structured discussion topic guide, with two fieldworkers conducting observations of the process and taking notes of the discussion. All fieldworkers were female university graduates with experience in social science and/or public health. In total, 12 FGDs were held, two with each of the homogenous groups A, B, C and D; and four with heterogeneous groups i.e. combinations of women from the four different groups (AB, CD, AD, BD) purposively chosen to assess whether their different experiences sparked interesting discussion, for example between those women who received blood tests or did not, those that received different types of anti-malarials, and those that received blood tests with or without then receiving anti-malarials.

All FGDs were conducted in the local language (Twi) and, with the informed consent of the participants, digitally recorded. Because Twi is predominantly a spoken language, translation and transcription into English were undertaken concurrently and these transcriptions were coded and analysed using NVivo version 8 [20]. A content analysis approach was used to identify themes based around the women's experiences of ANC and their involvement in the project [21]. The issues in the FGD topic guide included expectations of ANC visits, experiences during ANC attendance, reasons for repeat visits, and experiences with drugs and blood tests. Since women within the groups were of mixed gravidae, the reported differences in experience at ANC between last and previous pregnancies were used as a starting point to identify themes important to users of the current strategies under investigation. These were then used in conjunction with the FGD topic guide to further refine relevant themes.

### Ethical clearance and confidentiality

Ethical approval for this study was granted by the Committee on Human Research, Publications and Ethics, Kwame Nkrumah University of Science and Technology, School of Medical Sciences, Kumasi, Ghana; and by the ethics committee of the London School of Hygiene & Tropical Medicine. Verbal informed consent was sought from all participants and recorded before the start of all focus group discussions. During transcription, any names were replaced with codes to ensure anonymity and digital recordings were deleted once transcription and translation were completed and quality approved.

## Results

Using the comparison of current versus previous pregnancies amongst multigravidae in the FGDs as a starting point, three main themes were identified: (i) health benefits as a motivating factor for attending ANC, and as a positive experience of being in the project; (ii) services received; and (iii) health worker attitude. For assessing the relative acceptability of IST and IPT as strategies for controlling malaria in pregnancy, 'drugs received' and 'blood tests received' are discussed as distinct themes separately to the 'services received' theme.

### Health benefits

In the discussions around expectations of ANC, health emerged as a strong motivating factor for the women to attend ANC. The most frequently mentioned reason for ANC attendance was wanting to know if there were any problems for themselves or for the baby with specific concerns about avoiding adverse events, in particular losing the baby.

*"If you are a lady and you get pregnant, you must ensure the baby is healthy and so we go to the ANC expecting that if there is any problem anywhere the midwives would be able to tell us and treat us." *(Woman X, Group C1)

*"Some people do not go to the ANC and lose their babies as a result. I wanted to prevent that from happening to me. That's why I went to the ANC - I did not want to get any problems for myself." *(Woman D, Group A2)

The majority of the comments about the health of the baby related to knowing whether it was correctly positioned and what to do if it wasn't. Regarding their own health, some of the women who had already had other children mentioned that they went to ANC to try and avoid some of the problems they had experienced during previous pregnancies such as stiffness and high blood pressure. The primigravidae women wanted to find out about the changes that were happening to their bodies and to receive counselling on how to give birth safely. A number of women were concerned about non-specified "diseases" and wanted to find out if they had any so that they could be treated appropriately. Another common point of interest was in knowing the right kind of foods to eat during pregnancy to gain strength and energy for themselves and the baby.

Many of the multigravidae were keen to talk about the improvements in their own health and that of their babies that they perceived to have experienced during their most recent compared to previous pregnancies. Amongst this group, the vast majority felt that they were healthier and had a smoother delivery with this pregnancy compared to previous pregnancies. Positive comments were also made about their babies being strong, with one woman proudly showing the rest of the group:

*"Look my baby is less than 3 months old but see how fat and healthy she looks. The drugs and by means of God's grace our babies look very healthy." *(Woman A, Group D1)

However, these perceptions were not universal; some women did not notice any difference and one said that she had a more difficult pregnancy.

### Drugs

All women reported receiving many different types of medicine at each ANC visit and while some of the women described these drugs by name, others distinguished the different types by colour and size. Drugs that were commonly named by the women included multivitamins and B-complex, blood tonic and fesolate (iron tablets), while those who used colour as a descriptor talked of the green, brown or red pills (green and brown would normally be iron tablets, red normally multivitamins). Some women referred to anti-malarials by name, i.e. SP, Fansidar or artesunate-amodiaquine, others used a description e.g. three white tablets taken all together at the ANC (for SP), or the yellow and white pills (for AS-AQ).

Women in three of the FGDs mentioned differences in the drugs they had received at their ANC visits for previous pregnancies and the most recent one. Two women from one FGD group mentioned "three white tablets" (SP) that they had not received before; however, in the other two groups the discussions among the multigravidae relating to differences in drugs were about differences in the amount and type of medicines that could be classified as 'routine drugs' (as opposed to specific anti-malarials). The fact that in the majority of the groups the multigravidae women did not mention drugs in their discussions about differences between their pregnancies suggests either that these women did not identify that they had received different anti-malarial drugs or that these differences were not as important to them as other differences they had noticed during their last pregnancy. For some women it may be true that there were no differences in the anti-malarial drugs they received, namely those in the SP-IPT arm who could have received IPT in other recent pregnancies as routine practice. However, most of the women did receive different anti-malarial drugs, or at least according to a different strategy, during their recent pregnancy as part of the RCT. These findings could suggest either that the women don't know what the different drugs are for (and so could not distinguish that they had been given a different anti-malarial, or no anti-malarial), or that they know what the drugs are for but are not concerned about (or don't feel the need to discuss) the changes to the anti-malarial regime. From the comments made during the discussion it seems more likely to be the latter as most of the women seemed to have a reasonable idea what the various drugs they were given were for. Quite a few made specific mention of anti-malarial drugs, either providing a description or their actual name; when shown samples of SP and AS-AQ they recognized them and could say they were for malaria - either treatment or prevention.

*"The B complex and multivites were for good health of our babies, healthy appetite. And I believe the white ones were anti-malarial." *(Woman A, Group AB1)

However, some women did not seem so sure about the function of the different pills, but were nonetheless pleased with the perceived effects. When asked what the drugs were for, the women sometimes answered in terms of what effects they saw, rather than the biomedical reasons for taking the drugs during pregnancy. These responses give interesting insights into the perceptions of non-traditional medicines.

"*The medicine I took made me strong so I could do a lot of things whilst pregnant" - "Could you tell which ones made you strong?" - "No" - "Did you like the drugs anyway?" - "Yes" *(Woman E, Group A1)

*"I was told the green and red drugs performed the same function. My stool colour became dark after taking the green tablets and it was the same after taking the red tablets so I realized they were indeed the same in nature" *(Woman X, Group D2)

On the other hand, several side-effects and complaints associated with taking specific drugs were reported, either prompted or unprompted during the discussions. There were a small number of women who reported unpleasant experiences with AS-AQ or SP such as dizziness or weakness (AS-AQ), or vomiting (SP). However, with one or two exceptions where the women reported severe side-effects and refused to take the drugs again, most women said that they would still take the drugs due to the benefits that they associated with them:

*"The yellow and white tablets [AS-AQ] almost killed me. When I took it I became very weak and I couldn't do anything... Ever since I've become afraid of it." *(Woman A, Group BD1)

*"If you really want to get well, you don't dwell on the fact that the medicine is bitter because you know that it will make you strong" *(Woman A, Group AB1)

A small number of women given an anti-malarial said that they recovered before completing the course and so they did not continue taking the drugs, but this was not a commonly reported occurrence.

There were also some cases where women reported not finishing all of the routine drugs they had been given for a variety of other reasons. For example, some women said that the nutritional supplements had the effect of making them feel hungry so if they didn't have enough money for food they did not take them. A couple of women mentioned that they did not want to take all of the drugs because they were worried that their baby would grow too big. This suggests some interesting perceptions about the effect of drugs and taking drugs during pregnancy. The women implied that by taking all of the drugs the baby would grow to be relatively large, and this could lead to a difficult delivery.

Despite the reported side effects and concerns, there were more positive comments on the effects of the drugs than negative. For example, increased appetite was seen by most women as positive because it led to increased food intake which resulted in more strength and energy for carrying on with their normal jobs. Other positive comments included that some of the drugs helped them to sleep well and made them urinate a lot. This was seen as advantageous as it helped to flush out illness or impurities. Indeed, many of the women reported that the receipt of drugs was one of their main motivations for going to ANC and it was their responsibility to take them for their own good:

*"There are some people who refuse to take the drugs given to them at the ANC. When they encounter any problems, they blame the midwives forgetting that it is their fault." *(Woman B, Group A2)

### Blood tests

Amongst the multigravidae, only two women made specific comments about their most recent versus previous ANC experience regarding tests. One commented that the blood test and treatment for malaria parasites (locally referred to by the abbreviation 'MPs') was different to their previous pregnancy; the other observed that they had previously had urine tests at each visit which they did not experience as often this time. This lack of specific comment reflects the general reaction among the women that the blood tests were not a 'stand-out' part of their ANC experience. That is not to say that they did not have opinions on the blood tests when specifically asked. Most of the women acknowledged that the tests were painful but they were generally perceived to be acceptable as long as they knew what the test was for. However, while some of the women were able to be specific and named malaria (or MPs), anaemia, or HIV as the reason for the blood tests, many of the women were more vague saying that the tests were for 'diseases'. Despite this lack of clarity on disease specificity, it was clear that most of the women liked to know whether or not they had any diseases so that if they did they could be treated appropriately. Likewise, the reassurance gained from a negative test result was important. As with the drugs, there were some women who made it clear that they were only agreeing to the blood tests for the good of their baby - it seems they would otherwise have refused. However, when specifically questioned about how the repeated 'pricks' made them feel, again the women did not seem overly concerned.

"*Even though it was painful I liked it because *[I] *was investigated." *(Woman G, Group BD1)

*"Had it not been for the health of my baby, I would not have agreed to the pricking" *(Woman B, Group AB1)

*"Yes *[it is painful], *but even the delivery is more painful than the pricking" *(Woman F, Group CD1)

While most of the women said they were happy to have tests so that they could find out about any diseases that they might have, when asked if they had been told the results of the tests, most of the women said they had not. However, they often followed that by saying that they had received drugs e.g. for malaria or been told to eat more as they didn't have enough blood (anaemia), or the result had been written in their maternal health books. These data suggest that the ANC staff rarely provide verbal feedback on test result but, based on the actions taken (or on the information written on their cards) the women are happy to make assumptions and come to their own conclusions about the test results.

In addition to the lack of objection to the blood tests, which suggests indirect acceptability of the IST strategy, a small number of women made comments which demonstrated a more direct acceptance; namely that they preferred to know the results of tests before being given a drug:

*"It is good because the results tell the kind of drugs to be given. I prefer to go to the lab and be told the results and given drugs than just given drugs without any results. It should continue*." (Woman B, Group CD1)

### Other services received

Aside from the discussions around drugs and tests received at ANC, there were a number of comments about other services received, some of which related directly to being in the trial rather than the different strategies *per se*. For example, free ITNs were given to all women who enrolled in the trial and a number of the women said they had heard about the project from friends and reported that getting a net was one of their motivations for attending ANC. Several of the multigravidae highlighted this as an important difference between their most recent and previous pregnancies. They also attributed health benefits (e.g., feeling stronger and having heavier babies) to having slept under an ITN.

In addition, as part of the RCT, local community drug distributors were recruited to follow-up the women in their communities. Their responsibilities included: collecting socio-demographic and household level data; reminding participants of their next ANC appointment; checking that drugs were being taken correctly; and investigating whether or not the ITNs were being used. These follow-up visits were frequently mentioned by the multigravidae as being an important and positive difference to the services that that they had received during previous pregnancies. It seems that this extra level of attention and contact was highly appreciated, especially by those that lived far from the health facility.

*"I like the fact that I was being visited regularly. One day I was at home when I was suddenly visited. They treated me very nicely. I did not get that during my previous pregnancy. They even checked to see if I was sleeping under my net"*. (Woman C, Group D2)

Three of the multigravidae also mentioned that they were impressed with the fact that the services provided at the ANC clinic were free which meant that they were able to attend more regularly. However, while some of the multigravidae may have perceived that this was associated with the trial, in fact following a policy change implemented in July 2008, ANC services had been provided free to all pregnant women in Ghana via the National Insurance Scheme. A number of the other women also commented on being impressed with receiving many free services. For example, other things mentioned were ultrasound scans, which they liked because it showed them the position of the baby and in some cases the gender. Others reported being given food or drink during their ANC visits while they waited in the queue, and water being available to take the drugs at the health facility.

Positive comments were also made about the advice that they were given during ANC. For example, they appreciated being told the right food to eat, the correct way to lie to ensure the baby was in a good position, how to deliver safely and things to do at home after the birth.

### Health worker attitudes

Many comments were made by the women about being treated 'warmly' and 'with care' during their ANC visits, referring to both the project staff and the midwives. Other comments related to the 'patience' and 'tolerance' of clinic staff, and having a fun time at ANC with prayers and singing at the start of the session. The warm attitude of the midwives also encouraged them to return for repeat visits, reporting that people like to go where they are treated nicely. One of the women's comments suggested that the attitude of the health workers was the most important aspect of their satisfaction; the drugs and other tests were the same as a previous pregnancy, but the extra care and attention was something different this time:

*"I think there is a difference because *[at] *the previous hospital we were only checked and given drugs, but in this last one the nurses really took their time and conversed with me and told me the kind of food to eat and the things to do when I come home. I think this was better"*. (Woman C, Group CD1)

Not everybody had such a positive experience and there were some reports of being shouted at. For example, one woman reported that when they complained about waiting a long time for their drugs the midwife got angry and wouldn't deal with them any further. However, although some negative comments were made these were actually very few, and tended to refer to other peoples' experiences that they had heard rather than their own.

*"Some people complained of a midwife who shouted at them when they were delivering their babies. She did not treat me this way, she was nice to me." *(Woman B, Group A2)

Several comments were made at various points throughout the discussions, which suggest that the health workers are respected and viewed as figures of authority and the women effectively do what they are told. The ways in which the women talk about their overall acceptance of whatever drugs and tests they are given reinforces this impression and suggests that they have a lot of trust in what the midwives are telling them and so they follow their advice/instructions.

*"I was strong throughout my pregnancy because I did everything I was told to do." *(Woman X, Group D2)

*"I did not know the reason for giving me the white drug. But I took it." *(Woman A, Group A2)

## Discussion

The themes that emerged during the discussions among women who had experienced other pregnancies were the same as those that emerged during the general discussions involving all of the women about IST and IPT and their overall experience of ANC whilst participating in the trial. That is, the health of themselves and their babies was a key motivating factor for ANC attendance among all groups and the services received and the attitude of the health workers were important features associated with satisfaction with those services.

In general, there was little to distinguish the perceptions and reported experiences among the women enrolled in the different arms of the trial. For example, women in the SP-IPT arm said that they had experienced several blood tests and contributed to discussions about these tests despite most likely only having had a single blood test at their first ANC visit and thereafter receiving SP presumptively. A number of women in the SP-IPT arm also mentioned that they had taken AS-AQ, and could provide comments on their experience with this anti-malarial. However, while these women should not have received AS-AQ as part of their involvement in the trial, if they had complained of malaria symptoms then they would have been tested and treated with AS-AQ if found to be parasite positive, as per national policy; SP is reserved for preventive treatment only. For the women involved, it may be that the underlying reasons for tests and treatments were not entirely clear and the overall differences in experience among the groups were so subtle that in the discussions of their experiences it was difficult to distinguish which group had been involved in which arm of the trail. This would perhaps explain why no strong differences in opinion were observed among the different groups.

The findings of the study did not always match with what the study team had expected to be important for the women. For example, it was expected that women might have objections to repeated finger pricks for blood tests, especially if they did not always result in any treatment.

However, the results have shown that this was not a major issue for the majority of women; even though the tests are painful the women said that they were prepared to accept them for the sake of their health. Likewise, their knowing that they did not have any problems after having the first test motivated them to have the repeated finger pricks. This acceptance probably reflects the trust that these women have in the midwives and the advice that they give. Similarly, it was expected that the participants may have had complaints about seeing other women receiving different drugs or treatment at the ANC; however this was rarely raised as an issue. Where it was discussed, some of the women said that they hadn't seen what other women received, others thought that they all had received the same treatment, while others had noticed differences but said that they didn't mind that they had all received different things as they believed they may all have had different ailments bothering them or be at different stages in their pregnancy.

These data suggest that even though the different strategies seem quite distinct to the study team, i.e. depending on which arm of the trial a woman is in she will either have a blood test at each ANC visit or not, and receive SP to take at the health facility or AS-AQ to take at home or nothing, in reality these differences become diluted by other events occurring at ANC. Such findings seem to imply that women aren't always aware of the full details of what they are being given; this may be in terms of the names and functions of all of the different drugs, or the reasons for and results of the blood tests. However, even if the women could not report what specific drugs or tests were for in a strict biomedical sense, many of them had their own rational explanations. For example, some of the routine drugs 'gave them more blood' or drugs that made them urinate a lot were considered good because they flush out disease. These results agree with the findings of the acceptability studies that were conducted alongside trials of intermittent preventive treatment in infants (IPTi) in Tanzania and Mozambique; mothers accepted IPTi delivered at the same time as routine vaccinations due to a perception of both services being 'generally beneficial', without necessarily being able to be provide specific details of the interventions [22,23].

However, it may be that the acceptance of variation in what women receive at ANC would have been different if some had received nothing at all and others had received something. In this setting the women are already receiving many routine drugs so adding in different anti-malarials is perhaps not particularly noticeable. However, if some women had received ITNs and others hadn't then comments made during the discussions suggest that there would have been more complaints.

Overall, it seems that the specific issues around the drugs and blood tests were less important to the pregnant women attending ANC in this study than their perceptions of the general health benefits and their experiences of the quality of services received. For example, most of the unprompted discussion revolved around an appreciation of the advice and counselling received from the midwives (and project staff), and the friendliness and patience of staff. Health was a strong motivation for attending ANC, both for themselves and their baby. On the whole, their personal perceptions of their last pregnancy were that the services they had received at ANC had made them strong, and led to smoother deliveries and heavier babies. There was no apparent difference between women in the different arms of the trial, suggesting that from the women's point of view both strategies are equally acceptable. The findings that ANC is perceived as important for the health of their pregnancy and that they have a strong sense of trust in the midwives is supported by findings from other studies [24].

Certain positive aspects reported by the women are undoubtedly related to being involved in a trial; for example, the free ITNs and home visits. Being involved in a trial *per se *may also have a positive effect on perceptions of the services received [25]. It is also possible that the attitudes of health workers may have been better in trial facilities due to the extra help that the project staff provided for the midwives. However, comments on polite and patient health workers were not universal, indicating that not all midwives were positively influenced by being involved in the project. Nonetheless, care must be taken in interpreting the results of this acceptability study, as the experiences of the participants during implementation of the various strategies under trial conditions may be noticeably different to their experiences of implementation within the routine ANC.

The project staff was present on ANC clinic days during the trial, conducting the RDTs and dispensing anti-malarial drugs as appropriate according to the arm of the trial the woman was in. However, all other activities of the ANC sessions, such as the health education talks, HIV counselling and testing, and urine dipstick tests, were conducted as usual by the midwives. One of the main differences should IST be introduced into routine ANC as a replacement for SP-IPT would be the need for regular RDTs to be carried out. Currently, all diagnostic tests for pregnant women in the facilities that were involved in the trial are conducted by a laboratory technician, with the exception of urine dipstick tests that the midwives do in the ANC. In the larger health facilities, an ANC clinic day is often arranged with 'stations' which the women move between after a group health education session to receive the different services such as weighing, blood pressure, routine drugs and SP-IPT. IST with a RDT could be introduced into this system with little impact on the time that the women had to be at the clinic. In the smaller facilities with less staff, introducing IST for the midwife to implement may mean that women have to wait longer than if they received SP-IPT. Thus, it is possible be that the acceptability of the two strategies under routine conditions will vary according to facility size or type. However, it is likely that in facilities with a laboratory technician, the technician would conduct the RDTs along with other routine laboratory assessments so there would not be a noticeable change for the women between the two strategies since they would be sent to the laboratory anyway, regardless of the strategy.

An increased workload for the midwives in the absence of the study team and possible stock outs of drugs or RDTs could impede on the perceived quality of service received. Negative health worker attitudes towards clients is a common theme in qualitative studies of experiences of health care in sub-Saharan Africa [24,26], and it is possible that the presence of the trial team resulted in changes to health worker practice that might not be maintained in non-trial conditions. In addition, as was found in this study, the perceived quality of ANC services is a strong motivator (or barrier) to attendance in a variety of settings [24,27,28]. Thus, maintaining positive health worker attitudes and the availability of high quality services under routine conditions remains a challenge, requiring on-going supervision and improvement and these factors will be critical to the success of any maternal health intervention. The cost of ANC is another commonly reported barrier to ANC attendance and the lack of fees for the services received by women in this trial was considered as another positive aspect of their ANC experience. All maternal health services are now free in Ghana via the National Health Insurance Scheme, with indications that this is increasing ANC attendance [29]. Provided the scheme continues, this is likely to be a facilitating factor to the success of future maternal health interventions.

In addition to being participants in a trial, it is also important to highlight the fact that the women who attended the FGDs may be different to those that didn't; even though there was some element of randomization to the selection process, not all of those selected then turned up. Similarly, women were excluded whose babies did not survive the pregnancy, women who may have expressed more negative views of the trial. However, the aim of FGDs is to build a picture of the range of opinions on the topic under investigation. In this instance, after conducting 12 FGDs 'saturation' was reached with similar perceptions, views and experiences being expressed across the groups and no new information being provided. While these data are not, therefore, generalized to all of the trial participants, they do provide an insight into the issues that many of the women themselves perceived to be of importance in their experiences of the ANC services and malaria prevention strategies received during the trial.

The current treatment regimen for AS-AQ is two tablets of artesunate and two tablets of amodiaquine in the morning and evening for three days. This is considerably more complex than the treatment or prevention regimen for SP which involves a single dose of two or three tablets and can thus be taken as directly observed therapy (DOT), as recommended for SP-IPT. This has implications for adherence to IST-AS-AQ. However, the little evidence available in the published literature on adherence to AS-AQ suggests that this barrier can be overcome through appropriate information given by providers [30-32]. During an RCT of alternative anti-malarials for the treatment of malaria in pregnancy, Tagbor *et al *[30] found that despite a high prevalence of minor adverse events, 96% of women completed the 3-day course of AQ or AQ-SP combination. Such findings are supported by this study; a small number of women given AS-AQ said that they recovered before completing the course and so they did not continue taking the drugs, but this was not a commonly reported occurrence. Nevertheless, adherence to AS-AQ will need to be monitored as a potential operational barrier if the IST strategy is implemented on a large-scale [33].

In addition to understanding the acceptability of the strategies from a user perspective, it is also necessary to understand the provider perspective, including implications for implementation. A more detailed analysis of provider experiences and preferences of IST and IPT will be reported elsewhere, along with an exploration of factors contributing to implementability under routine (non-trial) conditions.

## Conclusion

Overall, both intermittent screening and treatment and intermittent preventive treatment seem to be equally acceptable to pregnant women as strategies for the control of malaria in pregnancy in this setting. In contrast to the trial team's perspective of differences between the two strategies that may influence the acceptability, including frequency of blood tests and receiving different drugs, the women were more concerned with the quality of services received, in particular the polite and patient attitude of health staff, and the positive health implications for themselves and their babies.

Although these are findings from experiences in a controlled trial setting, the study provides an insight into the potential motivating factors and barriers to delivering IST or IPT under routine ANC conditions. Encouragement of respectful communication between midwives and their clients can help to reinforce the trust relationship that exists. Good quality services at ANC are necessary to promote high coverage and to achieve actual and perceived health benefits for mothers and their children.

## Abbreviations

ANC: antenatal care; AS-AQ: artesunate-amodiaquine; DOT: directly observed therapy; FGD: focus group discussion; IPTp: intermittent preventive treatment during pregnancy; IPTi: intermittent preventive treatment for infants; IST: intermittent screening and treatment; ITN: insecticide-treated net; MoH: ministry of health; MP: malaria parasite; PMTCT: prevention of mother to child transmission of HIV; RCT: randomized controlled trial; RDT: rapid diagnostic test; SP: sulphadoxine-pyrimethamine; WHO: World Health Organization.

## Competing interests

The authors declare that they have no competing interests.

## Authors' contributions

JW, CJ, LS, HT and DC devised the study design and objectives. LS, CJ, NAB, ROA, GDA, and JW contributed to data collection, analysis and interpretation. LS did the analysis and wrote the first draft of the manuscript. All authors read, commented on and approved the final manuscript.
